# Effects of digital and non-digital parental distraction on parent-child interaction and communication

**DOI:** 10.3389/frcha.2024.1330331

**Published:** 2024-05-21

**Authors:** Souhir Chamam, Alexia Forcella, Nadia Musio, Florence Quinodoz, Nevena Dimitrova

**Affiliations:** ^1^ Faculty of Social Work (HETSL | HESSO), University of Applied Sciences and Arts Western Switzerland, Lausanne, Switzerland; ^2^ Institute of Psychology, University of Lausanne, Lausanne, Switzerland

**Keywords:** screen, technoference, toddlers, interaction, language

## Abstract

Technoference, namely parental screen use in the presence of a child, is a widespread phenomenon that has negative effects on parent-child interaction and communication. When parents use screens around their children there are fewer interactions and parents are less contingent and responsive to the child. Additionally, children show more negative behaviors, such as whining, frustration, and outbursts. Communication is also affected—parents speak and gesture less towards their children and, in turn, children are less likely to develop their language abilities. It remains unclear, however, if parental distraction due to screen use affects parent-child interaction and communication more negatively compared to non-digital parental distraction. Fifty-two parent-child dyads (mean child age = 22 months, range 12–36 months) first played for 5 min (Time 1); then (Time 2), the parent was asked to fill out a questionnaire on a tablet (screen condition), on a printed form (paper-pen condition) or was not interrupted (control condition). Interactive quality was assessed at Time 1 and Time 2 using the Coding Interactive Behavior scale. Communication was assessed by coding the number of word tokens and types during Time 1 and Time 2; child gestures were also coded. Results revealed that when parents were distracted—either by the paper-pen or the screen questionnaire—the quality of the interaction significantly deteriorated (*p*_s_ ≤ .01) and the quantity of parental communication significantly declined (*p*_s_ ≤ .012). Importantly, the nature of the distraction did not matter: there were no significant differences between the paper-pen and the screen distraction conditions across Time 2 (*p*_s_ ≥ .59). Findings suggest that parental distraction matters for the quality of interaction and the amount of communicative bids, independently on whether parents were distracted by a digital or non-digital activity. These findings likely relate to complex factors related to young children's experiences and habits with parental screen use.

## Introduction

Young children rely on interactions with their parents in order to learn and develop their social and emotional skills. When parent-child interactions are disrupted—such as when the parent is being distracted by using a screen—the interaction is negatively affected. In the current study, we test how different parental distractions may disrupt the quality and quantity of interactions between parents and their toddlers. Specifically, we ask whether parental distraction from a screen activity leads to lower quality of the interaction and to less communicative exchanges between the parent and the child than parental distraction from the same activity on paper-pencil.

### Technoference

Digital devices are ubiquitous: people across all ages, all cultures and all socioeconomic backgrounds are using digital media on an everyday basis. For smartphones only, 82% of French families (1) and 97% of the Swiss population (2) own at least one device. Digital devices are not only widespread; they are also highly used. Accordingly, French adults use digital media for 5 h/day [France (3)], whereas this duration almost doubles for American parents [i.e., 9 h/day (4)].

Yet, when a parent uses a digital device in the presence of a child, the nature and quality of the parent-child interaction are impacted (5). In fact, interactions are frequently interrupted when parents use a screen technology. Radesky and colleagues were the first to operationalize parents’ level of device use during parent–child interaction as “the extent to which the primary focus of the caregiver's attention and engagement was with the [digital] device rather than the child” (2014, p. 845). Later on, McDaniel (6) coined the term “technoference” or “technology interference” to describe the situations when digital media intrude and interrupt parent-child interactions and communications. In an American survey, 68% of parents indicated that they feel distracted by their smartphone when spending time with their children (7). Such self-reports are supported by systematic observations: 73% of parents have used their smartphone while in a fast food restaurant with their children (8).

Given that technoference is not an isolated phenomenon, studies have examined the implications of technoference on children. Beyond issues related to safety concerns (9), there is emerging evidence that parental screen use around children might negatively influence child's development. A particular emphasis is put on young children (0–3 years) given the importance of parent-child interactions on early psychological development.

### Effects of technoference on parent-child interactions

Interactions between parents and infants play a crucial role in supporting various aspects of the child’s development (10). These interactions enable young children to develop their social awareness through continuous and mutually responsive exchanges with their parents (11, 12). Additionally, sensitive parent-infant interactions lay the groundwork for forming secure attachment bonds (13, 14). As a result, the children’s growing social awareness and attachment provide the basis and incentive for them to explore and learn about the world, foster healthy social and emotional growth (15), acquire language and communication skills (16, 17), gain insights into themselves and others, and establish positive social connections.

A crucial aspect of the quality of parent-child interactions is the ability of parents to detect, acknowledge and respond to child's behavior and communicative bids [i.e., parental sensitivity (18, 19)]. There is broad consensus that parental sensitivity is crucial for child development (20). However, when parents’ focus of attention shifts to a digital device instead of the child (i.e., technoference), their ability to be sensitive and responsive to their children is negatively impacted.

Research shows that when parents use screens in the presence of their child, there are fewer interactions (21, 22) and parents are less contingent and responsive to the child’s behavior (9, 23–27). For example, studies looking at parents in public places show that lack of sensitivity to the child, such as not noticing signs of emotional distress, increases in parents who use a smartphone, compared with those who don't (28, 29). A similar finding, but relating to duration of use, is reported in studies by Tharner and colleagues (30) and Wolfers and colleagues (31). In these studies the authors show that the longer parents use their smartphones, the less sensitive they are to their child. Additionally, technoference is related to increases in the number of conflicts with the child, the number of negative behaviors towards the child (8) and the dissatisfaction of the time spent with the child (21).

Parental screen use during parent-child interactions also affects the child. Technoference is associated with both more internalized behaviors, such as whining or pouting, and more externalized behaviors, such as agitation, frustration and outbursts (32–34). Children show more negative affect and less positive affect when their mother uses a screen during interaction (35). In addition, children show more behaviors to attract their mother's attention during an interrupted mother-child interaction with a screen compared to an uninterrupted interaction (36). A recent experimental study shows that technoference affects infants’ physiological reactivity (i.e., increased heart rate), suggesting that this may be a stressful context (37).

### Effects of technoference on parent-child communication

In the first years of life, parent-child interactions provide the foundations of young children's communicative development. Stemming from the Transactional Theory in child developmental (38), the importance of face-to-face parent–child interactions in the development of communication in early childhood is widely recognized (39–42). Mounting evidence establishes direct links between the parental communicative input and young children's both verbal and non-verbal communication abilities. Namely, individual differences in maternal gesture rates correlate with their infants’ own gesture (43, 44) and parents who direct more speech to their children have children showing faster and larger vocabulary growth (45, 46).

There is empirical evidence showing that technoference affects parental communicative input towards young children (47). In a study with 6-year-old children, Radesky et al. (22) found that mothers who used their phone during the observation session spoke less and made fewer nonverbal gestures to their children (80% of the utterances and 61% of the nonverbal gestures compared to those who did not use phones). Importantly, findings of Reed et al.'s laboratory study showed that technoference affected vocabulary acquisition in toddlers. Specifically, 2-year-olds were less likely to learn a novel word taught by parents when they were distracted by a 30-sec phone call compared to peers whose parents were not interrupted (48). More recently, it has been shown that the amount of audible notifications parents report receiving per hour was negatively associated with infants’ vocabulary in controlled observations of 18- to 25-month-olds from New Zealand (49).

### The current study

Existing evidence shows that technoference is a widespread phenomenon that affects young children's development. Most research, however, has studied parental distraction by a screen compared to situations when parents are not distracted. While these studies highlight issues regarding screen use during parent-child interactions, it is clear that parents are oftentimes distracted in many other ways during interactions with their child, such as attending to another sibling or finishing cooking a meal. In order to determine the effect of parental distraction from using a screen, it is crucial to provide evidence from experimental conditions when parents are distracted from a similar but non-digital activity. To our knowledge, the empirical evidence on the differential effect of technoference compared to other parental distractions is very scarce. In a U.S. study on question-asking during parent-child interactions, Gaudreau et al. (50) found that only for information-seeking questions of parents—but not of children, nor for responsiveness to questions—distraction from a cell phone showed more negative impact than distraction from a non-digital activity. This finding points to the importance of controlling for parental distraction in order to clarify whether technoference affects parent-child interactions *above and beyond* non-digital parental distractions.

Accordingly, in this study we ask whether technoference affects the interaction and communication skills of parents and young children above and beyond parental distraction from a paper-pen activity. Based on the literature, we made the following hypotheses:

1. Both interaction and communication will be negatively affected when parents are distracted compared to when parents are not being distracted.
2. Both interaction and communication will be negatively affected when parents are being distracted in the screen condition compared to when parents are being distracted in the paper-pen condition, given that digital devices may be especially distracting to parents (23, 51).

## Methods

### Population

Fifty-two parent-child dyads were invited to participated in this study. Two dyads were excluded because of technical problems preventing from coding the data. The final sample consisted of 50 parent-child dyads; however, interaction scores are missing for one dyad due to disturbances during data collection. The G*Power software indicated that the obtained power for correctly rejecting the null hypothesis (1-β err prob) given an effect size of .25 and the study's sample size is .95.

Mean child age was 22 months (min = 12, max = 36, SD = 7.37 months); 26 girls (52%) participated. Mean parental age was 34 years (min = 27, max = 49, SD = 4.72 years); 45 mothers (90%) participated. Parents were predominantly married or in a couple (88%), mostly highly educated (67% held a university degree) and the large majority had a professional occupation (68%). Participants were recruited in the metropolitan area of a large French-speaking city in Switzerland by posting flyers in day-care centers and pediatric practices, by word-of-mouth, and through advertisement on social networks.

### Procedure

**Figure 1:**
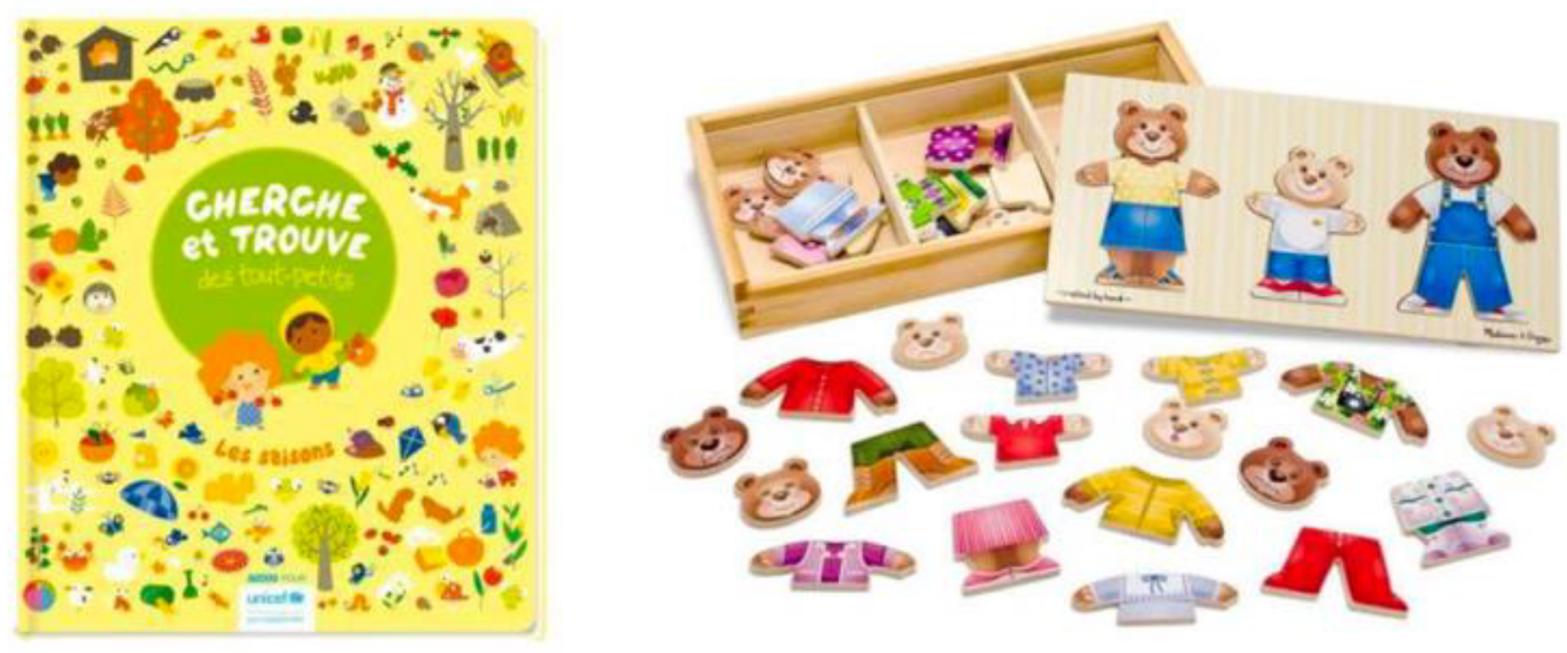
Materials used for the interactive play session.

Data was collected in a quiet laboratory room over the summer of 2021. Upon arrival of the parent-child dyads, an experimenter explained that the purpose of the study is to evaluate parent-child interaction (without mentioning parental distraction), answered eventual questions and obtained written consent from parents. Each parent-child dyad was invited to sit on a foam tile carpet on the floor; several cushions were supplied. A seek-and-find book as well as a wooden bear puzzle were provided for the interactive play session (see Figure 1). All parent-child interactions were videotaped by a static camera. Before leaving the room, the experimenter reminded participants that the interaction was being filmed and asked them to remain facing the camera.

All dyads were instructed to play for 10 min as they usually would do in a quiet room. Participating dyads were randomly assigned into one of three condition. In the first condition, parent-child dyads interacted for 10 min without distraction (“control group”, 16 dyads, or 32%). In a second condition (“paper-pen distraction”, 17 dyads, 34%), each dyad interacted for 5 min without distraction (Time 1), then was interrupted by the experimenter who asked the parent to fill in a demographic questionnaire on paper while continuing to interact with the child for additional 5 min (Time 2); the experimenter then left the room. In the third condition (“screen condition, 16 dyads, 32%), each dyad interacted for 5 min without distraction (Time 1), then was interrupted by the experimenter who asked the parent to fill in a demographic questionnaire on a digital tablet while continuing to interact with the child for additional 5 min (Time 2); the experimenter then left the room.

In all conditions, after ten minutes of interaction, the experimenter returned to the room and stopped the video recording. Parents were then invited to complete the demographic questionnaire either in the lab or at home. Each session lasted approximately 40 min. At the end of each session, participants were informed about the focus of the study on parental distraction and were given the possibility to retract their participation if they no longer agreed with the aims of the study; no participants retracted. All participants received a CHF20 gift voucher for a bookstore.

### Measures

#### Demographic questionnaire

A 13-item demographic questionnaire contained questions about the child (e.g., age, nationality, child’s place in siblings and dominant childcare arrangement) and the parent (e.g., age, gender, education and marital status).

#### Interaction skills

The interaction skills of the child, the parent and of the dyad taken together were assessed during the 10-minute observed play using the Coding Interactive Behavior scheme [CIB (52, 53); for French validation]. This tool codes parent-child interactions across three types of scales: child, parent, and dyad.

The child scale consists of items divided into three composite scores: social involvement, withdrawal/negative emotionality, and compliance; for the purposes of this study, we focus on the social involvement and withdrawal composite scores. The child social involvement composite score is coded on the following 9 items: child gaze/joint attention, positive affect, child affection to parent, alert, fatigue (reversed score), child vocalization/verbal output, child initiation, competent use of environment, and creative symbolic play. The child withdrawal composite score is coded on the following 4 items: negative emotionality/fussy, withdrawal, emotional lability, and child avoidance of parent.

The parent scale type consists of items divided into four composite scores: sensitivity, intrusiveness, limit setting, and negative mood; for the purposes of this study the two former composite scores are used. The parent sensitivity composite score is coded on the following 10 items: acknowledging child’s signals, elaborating on child's signals, parent gaze/joint attention, positive affect, vocal appropriateness/clarity, appropriate range of affect, resourcefulness, praising of the child, affectionate touch, and parent supportive presence. The parent intrusiveness composite score is coded on the following 4 items: forcing, overriding child's signals, parent anxiety, and criticizing the child.

The dyad scale consists of items divided into two composite scores. The dyadic reciprocity composite score is coded on the following 3 items: dyadic reciprocity, adaptation-regulation, and fluency. The negative state composite score is coded on the 2 following items: constriction and tension.

Each item is coded on a scale from 1 to 5, as follows: no manifestation of the item's behavior is observed (1), some manifestations are present but not frequent or constant during the interaction (3), manifestations of the item's behavior are frequent and constant throughout the interaction (5). Codes of 2 or 4 can be used to indicate a tendency towards a low (2) or high (4) level. Accordingly, higher scores indicated higher child social involvement, higher parental sensitivity, but also higher child withdrawal, and higher parental intrusiveness.

For the purposes of this study, the 10 min play session was divided into the first 5 min of the interaction (Time 1, no distraction) and the remaining 5 min of interaction (Time 2, no distraction for the control group, paper-pen distraction, or screen distraction). Each time (Time 1 and Time 2) was coded separately.

Coders were trained to use the CIB scheme by a licensed coder who obtained reliability with Ruth Feldman’s team. Once coders reached reliability with the licensed coder on a different video dataset, they coded the video data from the present study. A randomly selected 20% of the video data was double-coded; inter-rater agreement was 80%, indicating that both coders gave the same score or a score that differed by maximum one point at the 5-point Likert scale in 80% of the cases.

#### Communication skills

Verbal communication skills were assessed using the word tokens and word types produced by the child and the parent during the 10 min play interaction. Additionally, non-verbal communication skills were evaluated in children through the gestures they produced during the 10 min interaction.

Word tokens and types scores were based on the transcription of the speech produced by the child and the parent. Speech was transcribed following an adaptation of transcribing conventions from Hoff (54). Transcriptions were analyzed for total number of words produced during the interaction (i.e., word tokens, e.g., “go, go!” counts as two word tokens) and for total number of different words produced during the interaction (i.e., word types; e.g., “go, go!” counts as one word type) using the CLAN software. Word tokens and word types frequencies were extracted for each protagonist, i.e., child and parent. For the purposes of this study, each protagonist received a word tokens and types frequency score for Time 1 and Time 2.

We further coded for children's nonverbal communication skills during the parent-child interaction, following earlier work (55). Gesture was defined as a communicative hand (e.g., pointing at a ball, extending open palm toward a ball) or body movement (e.g., shaking head sideways to convey negation, extending arms sideways to convey airplane) that was directed to the parent and that did not manipulate objects, such as hammering a peg. All gestures were empty-handed with the exception of show gestures, during which the child brought an object to the parent's attention by holding it up. The frequency of gestures produced by the child was determined for Time 1 and Time 2 of the parent-child interaction session. A randomly selected 20% of the video data was double-coded; inter-rater agreement was 80%.

### Data analysis

The dependent variables in our analyses were the following: score for child social involvement during the interaction, score for child withdrawal during the interaction, total number of gestures produced by the child, total number of word tokens produced by the child, total number of word types produced by the child, score for parental sensitivity during the interaction, score for parental intrusiveness during the interaction, total number of word tokens produced by the parent, total number of word types produced by the parent, score for dyadic reciprocity during interaction, and score for dyadic negative states during interaction; all dependent variables showed a non-normal distribution. In order to answer our first research question, namely to determine whether parental distraction—independently of whether it is a paper-pen distraction or a screen distraction—alters parent-child interaction and communication, we performed a Wilcoxon signed-rank on the dependent variables between Time 1 and Time 2 for the paper-pen and screen conditions taken together. In order to determine whether parental distraction from filling out a questionnaire on a *screen* (i.e., technoference) alters parent-child interaction and communication above and beyond parental distraction from filling out a questionnaire on a paper-pen format, we performed a set of Kruskall–Wallis analyses on the dependent variables between the three experimental conditions (no distraction, paper-pen distraction, screen distraction) at Time 2. Last, for the dependent variables that showed significant differences between the three conditions at Time 2, we performed Mann-Whitney tests in order to obtain post-hoc comparisons.

## Results

### Does parental distraction affect parent-child interaction and communication?

Comparing parent-child interaction and communication between Time 1 (i.e., no distraction) and Time 2 (paper-pen or screen parental distraction) shows that parental distraction matters (see Table 1).

**Table 1:** Descriptive statistics (means; standard deviation scores in parentheses) for the dependent variables across the two assessment times (Time 1, Time 2) for the paper-pen and screen conditions taken together. The last column displays the *p*-values for the difference test between the two times.

		Time 1	Time 2	*p*-value
Interaction	Parental sensitivity	4.46 (.29)	3.76 (.55)	.001***
	Parental intrusiveness	1.44 (.45)	1.41 (.53)	.532
	Child social involvement	3.85 (.42)	3.43 (.52)	.001***
	Child withdrawal	1.28 (.58)	1.51 (.67)	.01**
	Dyadic reciprocity	4.46 (.70)	3.21 (.84)	.001***
	Dyadic negative states	1.34 (.54)	1.98 (.44)	.001***
Communication	Child word tokens	44.53 (37.60)	41.74 (41.63)	.350
	Child word types	21.50 (17.43)	21.06 (18.49)	.610
	Child gestures	9.06 (8.02)	7.63 (6.95)	.299
	Parent word tokens	389.82 (149.02)	278.49 (140.63)	.001***
	Parent word types	152.70 (48.36)	129.83 (48.63)	.012*

*p ≤ 0.05; **p ≤ 0.01, ***p ≤ 0.001.

Regarding the quality of the parent-child interaction, our results showed that all of the examined variables, except for parental intrusiveness, differed between Time 1 and 2. Specifically, when parents were asked to fill out a questionnaire at Time 2 (either on a paper-pen or screen format), the quality of the interaction was significantly altered compared to Time 1 when they were only instructed to interact with the child: parents were less sensitive to their children's communicative signals and needs (*z* = 4.595, *p* < .001), children engaged less with the parent (*z* = 3.233, *p* < .001) and also showed more withdrawal behaviors (*z* = 2.590, *p* = .01), and the dyads interacted in less reciprocal ways (*z* = 4.962, *p* = .001), showing more negative states (*z* = 4.737, *p* = .001).

In terms of communication scores, however, results showed that only parental speech changed between Time 1 and Time 2, such that parents talked less to their children—both in terms of word quantity (*z* = 3.641, *p* = .001) and word diversity (*z* = 3.079, *p* = .012)—when they were distracted by filling out the questionnaire (Time 2).

Taken together, the analyses showed that when parents were distracted by filling out the questionnaire—independently on whether it was on a paper-pen or a screen format—the quality of the interaction as well as the child-addressed parental speech deteriorated.

### Does technoference affect parent-child interaction and communication skills?

Having established that parental distraction alters parent-child interaction and communication, we asked whether the nature of the distraction—digital vs. non-digital—matters as well.

Results showed that, in terms of interaction quality, parental sensitivity, *H*(2, *n* = 50) = 17.977, *p* < .001, child social engagement, *H*(2, *n* = 50) = 6.379, *p* = .041, and dyadic reciprocity, *H*(2, *n* = 50) = 16.727, *p* < .001, as well as dyadic negative states, *H*(2, *n* = 50) = 10.567, *p* = .005, were significantly different across the three experimental conditions at Time 2 (see Table 2). Regarding communication, only parental production of word tokens showed a tendency towards a significance between the conditions, *H*(2, *n* = 51) = 5.838, *p* = .054. Mann–Whitney comparisons revealed that, for all of the dependent variables that showed significant between-condition differences, the no distraction condition always differed significantly from the paper-pen as well as the screen condition (*p*_s_ ≤ .04). Importantly, for none of the dependent variables, there were significant difference between the paper-pen distraction condition and the screen distraction condition (*p*_s_ ≥ .59). This last result reveals that the nature of the distraction—namely, from a paper-pen or a screen activity—does not matter for the parent-child interaction and communication.

**Table 2:** Descriptive statistics (means; standard deviation scores in parentheses) for the dependent variables at Time 2 by condition. The last column displays the *p*-values of the difference test between the three conditions.

		No distraction	Paper-pen distraction	Screen distraction	*p*-value
Interaction	Parental sensitivity	4.46 (.33)	3.77 (.51)	3.77 (.60)	.001***
	Parental intrusiveness	1.56 (.60)	1.35 (.44)	1.51 (.62)	.733
	Child social involvement	3.78 (.27)	3.37 (.60)	3.56 (.35)	.041*
	Child withdrawal	1.28 (.36)	1.37 (.49)	1.67 (.82)	.465
	Dyadic reciprocity	4.38 (.73)	3.28 (.79)	3.29 (.88)	.001***
	Dyadic negative states	1.45 (.53)	2.01 (.39)	1.95 (.52)	.001**
Communication	Child word tokens	50.44 (56.74)	34.94 (29.63)	45.82 (51.03)	.912
	Child word types	26.44 (24.40)	19.35 (16.57)	20.82 (19.52)	.753
	Child gestures	9.63 (8.55)	8.53 (8.04)	7.12 (5.83)	.805
	Parent word tokens	368.75 (116.92)	267.23 (140.02)	287.82 (148.76)	.054^†^
	Parent word types	148.25 (34.21)	131.06 (50.30)	125.76 (48.29)	.259

^†^*p* ≤ 0.1; *p ≤ 0.05; **p ≤ 0.01, ***p ≤ 0.001.

## Discussion

In this study we asked whether technoference affects the interaction and the communication between parents and their children during a 10 min play. Observing 50 parent-child dyads, we found that parental distraction matters for the quality of interaction and for the quantity of communicative acts, independently on whether parents were distracted by a paper-pen questionnaire or by a questionnaire on a screen.

### Effect of distraction on parent-child interaction and communication

Our first main result shows that when parental focus of attention is shifted from the child to another activity, the quality of interaction is negatively impacted. Specifically, when parents were distracted during the parent-child play, parents were less sensitive to children's communicative signals, children showed lower social involvement, and the dyads showed less reciprocity and more negative states in their exchanges. These findings align with previous theoretical and empirical work highlighting that children, especially young children, need the attention of their parents during moments of interaction and play. When parents pay attention to their children, they acknowledge and respond to the child's behavior and needs, which, in turn, contributes to the child's early social, communicative and emotional development (10).

Importantly, our study suggests that the fact that parents are not focused on the interaction with their child has a negative effect not only on themselves (i.e., parents being less sensitive to the child), but also on the child *and* on the dyad altogether. Precisely, from early on, children are able to detect that the parent is non-contingent and unsynchronized when being distracted, which results in the child being less involved in the interaction with the parent and more withdrawn, as indexed by behaviors such as sharing less joint attention, showing less positive affect, producing less verbal output, initiating less interactions, etc. Unsurprisingly, the parental distraction during the parent-child interaction also negatively affects the interactive quality of the dyad: dyads engage in less give-and-take synchrony, coordinate less levels of arousal and stimulation, show less smooth and fluent flow of activity and involvement and show higher levels of constriction and tension.

Our findings showed that parental distraction also affects the communicative exchanges between the parent and the child during the 10 min play. Specifically, parents produced less word tokens (i.e., measure of verbal quantity) and less word types (i.e., measure of verbal variability) in the conditions when parents were distracted. This result confirms our hypothesis according to which parental distraction negatively influences the communicative bids between the parent and the child. Interestingly, we did not find differences in the verbal or non-verbal communication that children were addressing to parents across the undistracted (Time 1) vs. distracted condition (Time 2). It is possible that when parents were distracted, children continued to produce communicative bids in order to regain the attention of their parent, thus maintaining a similar level of communication. This hypothesis remains to be further examined in future studies.

### Lack of effect of technoference on parent-child interaction and communication

Our second main result is that technoference, namely parental distraction due to using a digital device, does not affect parent-child interaction, nor communication more so than non-digital parental distraction. More specifically, we failed to found a difference between parental distraction due to using a screen compared to parental distraction due to a non-digital activity (i.e., paper-pen condition). This result is in conflict with our hypothesis—we expected that technoference will show a more pronounced negative effect on parent-child interaction quality and communication compared to the non-digital distraction, given that screens are especially distracting to parents (51). Why the lack of effect then?

A first explanation lies within the existing evidence. While the vast majority of studies on technoference point to negative effects, including on parent-child interaction and communication, these studies are either qualitative, thus failing to provide comparisons from experimental conditions, or compare conditions of parents using screens (i.e., technoference) to conditions of parents not using screens [i.e., paying attention to the child; for a review, see (25)]. Therefore, general conclusions about the negative effects of technoference have been drawn, although without examining whether these effects stem specifically from parental distraction *by screens* or simply from parental distraction.

A major strength of the present study is to provide comparisons of parent-child interaction and communication across three experimental conditions: undistracted parents, parents distracted by a non-digital activity (i.e., completing a paper-pen questionnaire) and parents distracted by an activity on a screen (i.e., technoference). Such comparisons suggest that parents are not more distracted by a screen than by another non-digital activity. Recent literature providing evidence from similar comparisons show similar findings. For example, comparing parent-child interactions while parents used a cell phone to parent-child interactions while parents completed a paper survey, Gaudreau et al. (50) did not find a difference in parental, nor child responsiveness. Similarly, while Abels et al. (23) show that when caregivers use mobile media they are less responsive to children's bids for attention, their findings indicate that this appears to also be true when caregivers are involved in other non-child-related activities.

Taken together, our results add to the existing evidence showing that parents do not seem to be more distracted when using a screen compared to other types of distraction. This might be so because screens are so ubiquitous in today's society, that both parents and children have become accustomed to such devices. Stockdale et al. (56) suggests that this could imply a form of self-regulation learning for the child. In the same vein, we can also assume that the parent becomes accustomed to and implements communicative strategies when using a screen during interactions with the child. This assumption could reduce the negative effects on the quality of the interaction.

It is also likely that digital devices prompt a joint attention phenomenon; namely, children could find screens attractive, thus increasing the likelihood of joining in the parent's focus of attention drawn from the screen—possibly more so than when parents are involved in a non-screen activity. There is evidence that when parents and children are co-viewing and especially when parent use this co-viewing as an opportunity for interaction, conversation and sharing (5), children do benefit from such screen use, including for their language development (57).

### Limitations

While the present cross-sectional study adds important findings to the existing literature, it also presents a number of limitations. First, the sample represents a limitation in terms of size and representativeness. It includes 50 dyads (considered a high number for observational studies) and is relatively homogeneous in terms of parent gender (vast majority of mothers), parent education (highly educated parents), and marital status (over-representation of married parents/couples). Second, ecological validity is affected by the experimental setting, which is not representative of everyday parent-child interactions in a natural environment. This may result in a motivational bias with regard to participation, with parents in difficulty with their child avoiding taking part in the study. This may also manifest itself as a social desirability bias through the desire to satisfy social expectations in terms of child rearing. Importantly, the type of parental digital distraction in this study (i.e., completing a questionnaire on a tablet) is different in many ways from the real world, everyday ways in which parents use screens in the presence of their children. Specifically, in our study, parental digital distraction did not include the personal or professional context of screen use, neither any emotional aspect in the nature of the digital distractor, such as for example when consulting work emails or responding to personal messages. In such naturalistic situations of screen use, parents are likely more compelled to use screens and presumably they are more absorbed in the screen use and distracted from the interaction with the child.

Last, parental gestures were not coded due to personnel shortage. It would have been interesting to have the coding of parents’ gestures in order to better understand certain effects observed in the interaction. In fact, several authors (58–60) have stressed the importance of the synchronicity aspect in the interaction, which implies a mutual influence between one and the other.

Drawing from these limitations, future studies are needed in order to provide better understanding of the effect parental use of screens might or might not have on young children. Importantly, longitudinal studies controlling for a number of significant confounds are truly needed in order to capture possible causal links. These include, but are not limited to, the level of immersion (61) or absorption (25, 30, 57) of the parent during screen use and children's everyday experiences and habits with parental screen use.

## Conclusion

This study suggests that technoference does disrupt parent-child interaction, but in similar ways than an equivalent non-digital parental distraction. The finding adds to the extensive literature on the importance of parental involvement for the quality of the parent-child interaction. It also allows to dedramatize a certain “moral panic” (62) surrounding screen use. More specifically, it suggests that it might not the use of the screen itself that is derogatory for the interaction; rather, it could the fact that the parent is kept away from the interaction, independently from whether it is a digital or a non-digital distraction.

## Data Availability

The raw data supporting the conclusions of this article will be made available by the authors, without undue reservation.
